# Internal dynamics of intense twin beams and their coherence

**DOI:** 10.1038/srep22320

**Published:** 2016-02-29

**Authors:** Jan Peřina, Ondřej Haderka, Alessia Allevi, Maria Bondani

**Affiliations:** 1 RCPTM, Joint Laboratory of Optics of Palacký University and Inst. Phys. CAS, 17. listopadu 12, 77146 Olomouc, Czech Republic; 2 Institute of Physics CAS, Joint Laboratory of Optics, 17. listopadu 50a, 77146 Olomouc, Czech Republic; 3 Dipartimento di Scienza e Alta Tecnologia, Università degli Studi dell’Insubria, Via Valleggio 11, 22100 Como, Italy; 4 CNISM UdR Como, Via Valleggio 11, 22100 Como, Italy; 5 Istituto di Fotonica e Nanotecnologie, Consiglio Nazionale delle Ricerche, Via Valleggio 11, 22100 Como, Italy

## Abstract

The dynamics of intense twin beams in pump-depleted parametric down-conversion is studied. A generalized parametric approximation is suggested to solve the quantum model. Its comparison with a semiclassical model valid for larger twin-beam intensities confirms its applicability. The experimentally observed maxima in the spectral and spatial intensity auto- and cross- correlation functions depending on pump power are explained in terms of different speeds of the (back-) flow of energy between the individual down-converted modes and the corresponding pump modes. This effect is also responsible for the gradual replacement of the initial exponential growth of the down-converted fields by the linear one. Furthermore, it forms a minimum in the curve giving the effective number of twin-beam modes. These effects manifest a tight relation between the twin-beam coherence and its internal structure, as clearly visible in the model. Multiple maxima in the intensity correlation functions originating in the oscillations of energy flow between the pump and down-converted modes are theoretically predicted.

Nowadays, parametric down-conversion (PDC) describing three mutually interacting optical fields^1^ represents the most common source of nonclassical light^2^. This is due to the natural pairwise character of the nonlinear interaction generating one photon pair at the expense of an annihilated pump photon. As the signal and idler photons in a pair are emitted together^3^, their properties, including polarization, frequency and wave-vector, exhibit strong correlations. These correlations occur at the level of the amplitudes of their wave function so that they have no classical counterpart. Entanglement gives unusual properties to photon pairs that lead to the violation of the laws of classical physics^4^, to quantum teleportation^5^ and to many other purely quantum effects. Photon pairs may also form intense beams, the so-called twin beams (TWB)^6^,^7^. In the TWBs, the quantum features of photon pairs are partly concealed due to their macroscopic character. However, tight correlations in the numbers of signal and idler photons remain. They represent the origin of sub-shot-noise intensity correlations^8^,^9^,^10^,^11^, which are useful, e.g., in ghost^12^ and quantum^13^ imaging. These intense TWBs are typically multimode and display an interesting internal structure^14^,^15^. We note that also single-mode intense TWBs can be obtained applying strong spectral and spatial filtering^16^. During the generation of intense TWBs, the dynamics of PDC is more complex^15^,^17^ and naturally allows for the back-flow of energy from the modes of the down-converted fields into the pump-field modes. As it has been experimentally shown in^18^,^19^,^20^,^21^, this results in a partial loss of coherence both inside the signal and idler fields and between them. As we show here, this partial loss of coherence reflects the changes in the internal ‘intensity’ structure of an intense TWB affected by the back-flow of energy. To provide a detailed insight into such a behavior we develop two models appropriate for intense TWBs endowed with multithermal statistics. They are based on the Schmidt mode decomposition^22^,^23^ applied to both spectral and transverse wave-vector domains where it provides the ‘natural physical bases’ for TWBs. These models represent an alternative to the previously developed theories based on quasi-monochromatic and quasi-plane-wave approximations^24^ as well as to models exploiting numerical solutions of the Maxwell equations and simulations^25^. Whereas the former model^24^ is not applicable for depleted pump beams, the latter one^25^ does not provide an insight into the internal dynamics of the PDC.

We note that the model developed here for twin beams may be useful also for other three-mode nonlinear interactions found, e.g., in acousto-optics^26^, electronics or opto-mechanics^27^. More general models can be developed for other nonlinear processes decomposable into three-mode interactions.

In the process of PDC, pump, signal and idler fields mutually interact in a nonlinear medium with an effective nonlinear susceptibility 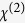. Assuming for simplicity scalar fields, the appropriate nonlinear momentum operator 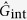 for PDC takes the form^1^,^28^,^29^:


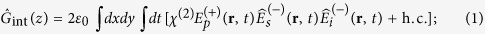



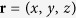
. In Eq. (1), 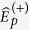 describes the positive-frequency part of the pump electric-field operator amplitude and 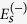 [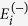] denotes the negative-frequency part of the signal [idler] electric-field operator amplitude. Symbol 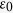 is the permittivity of vacuum and 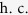 replaces the Hermitian conjugated term.

The momentum operator 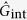 can be recast into its ‘diagonalized’ form approximately revealed by the Schmidt decomposition of weak TWBs. In this case, the so-called two-photon amplitude^30^, describing the common state of signal and idler photons, is calculated as a perturbation solution of the corresponding Schrödinger equation. The subsequent Schmidt decomposition provides the orthogonal paired modes that are the ‘genuine physical modes’ of the signal and idler fields. In addition, the Schmidt coefficients *λ* giving the quantum probability amplitudes of finding a given paired mode in the state are obtained (for details, see the Supplemental material^31^). Using these modes and coefficients both in the transverse wave-vector plane^23^,^32^,^33^ and in the frequency domain^22^, the momentum operator 
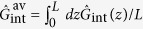
 ‘averaged’ over the crystal length 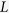 is obtained as:


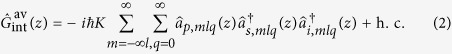


In Eq. (2), the annihilation (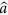) and creation (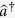) operators belong to the pump, signal and idler modes that form independent modes’ triplets. The common coupling constant 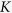 includes multiplicative factors quantifying the nonlinear interaction in the transverse wave-vector plane (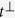) and in the frequency domain (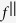) as well as the normalization to photon numbers (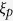); 
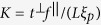
 [for more details, see^17^]. The overall pump-field amplitude 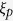 is derived from the pump-field power 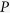, its repetition rate *f* and central frequency 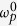 as 
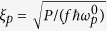
, where 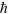 stands for the reduced Planck constant. It is assumed that the overall pump power *P* impinging on the crystal can be divided into individual pump modes indexed by 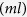 (transverse wave-vector components) and *q* (frequency components) proportional to the squared product 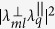 of the Schmidt coefficients that characterize the two-photon amplitudes in the transverse wave-vector plane and in the spectrum. Thus, an 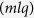-th mode of a classical strong pump field has its incident classical amplitude 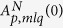 [corresponding to the mean value of normally-ordered quantum amplitude] given as 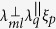.

The Heisenberg equations derived from the momentum operator 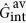 in Eq. (2) describe the evolution inside independent subspaces of the individual modes’ triplets 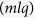. In what follows, we further pay attention to an arbitrary subspace 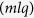 and omit its indices for simplicity. Before we address the nonlinear interaction in its quantum form, we first consider it as *a classical nonlinear problem*. The Schmidt decomposition provides the signal and idler modes in pairs in which they share a common Schmidt coefficient *λ*. As a consequence, the signal and idler field amplitudes (expressed in photon numbers) belonging to one pair are the same. Using this symmetry, the classical counterpart of the Heisenberg equations written for mean values of *the symmetrically-ordered* signal (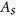) and pump (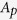) *operator amplitudes* attains the form [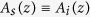, 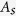 and *K* are assumed real]:


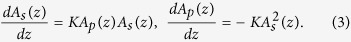


The integral of motion 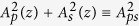 in Eqs. (3) allows us to find their solution by direct integration of the second equation in (3):


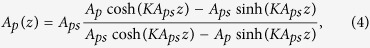



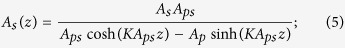



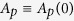
 and 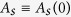. As equations (3) are written for the symmetric ordering of field operators, the incident pump, vacuum signal and vacuum idler amplitudes are equal to 
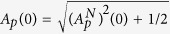
 and 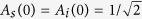. The solutions (4) and (5) are valid until the pump mode is completely depleted. This occurs at 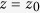 for which 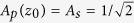:


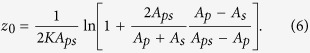


In the interval 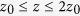 the flow of energy in the analyzed modes’ triplet is reversed, i.e. the pump mode takes back the energy from the down-converted modes. In this case, fields’ evolution is again described formally by Eqs (4) and (5), however, with *z* replaced by 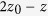. At 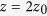 the modes’ triplet returns to the incident state and the dynamics repeats from the beginning.

To include the quantum statistical character of the signal and idler fields in multi-thermal PDC, we first propose *a model based on the generalized parametric approximation* (GPA)^34^. In this approximation, the pump field is taken as a classical field arising from the classical solution written in Eq. (4), i.e. it undergoes depletion. This removes nonlinearity from the original Heisenberg equations derived for the momentum operator 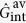 in Eq. (2). The resultant Heisenberg equations form a simple linear operator set of equations:


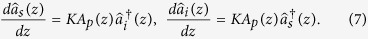


The solution of Eqs. (7) is found in the usual way:


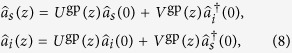



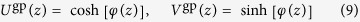


defining 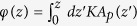. For the classical pump amplitude 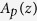 written in Eq. (5), we have:


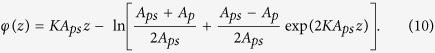


We note that the second term on the r.h.s. of Eq. (10) accounts for the pump depletion and so it goes beyond the usual parametric approximation. The solution (8) and (9) preserves the canonical commutation relations. As we will see below, though the model does not preserve the overall energy (the momentum operator 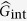 is nonconservative), it describes well coherence and mode structure of the TWBs.

To verify the validity of GPA, we develop in parallel *a semiclassical model* (SCM) that preserves the overall energy. In this model, we build the operator solution from the classical one written in Eq. (5) using the amplitudes related to the normal 
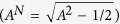
 and anti-normal 
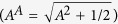
 ordering of field operators. This solution is obtained in the form of Eqs. (8) using the following coefficients


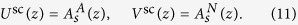


We recall that 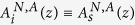. This model is valid for larger intensities for which the quantum description coincides with the classical one. Moreover, the model is appealing also for lower intensities as it preserves the commutation relations.

Pump depletion naturally limits the pump powers *P* for which an exponential growth of the number *N* of emitted photon pairs is observed^18^. The increase of the overall photon-pair number *N* slows down as the pump power *P* increases and finally a linear increase is reached (see Fig. 1). This change occurs for the pump powers *P* at which the individual pump modes containing the largest portion of the incident pump power (with the largest Schmidt coefficients) become completely depleted and begin to take their energy back from the signal and idler modes with whose they share the common modes’ triplets. This reflects the most important feature of the nonlinear dynamics of individual modes’ triplets in PDC: *the larger the mode’s incident pump power, the faster the nonlinear dynamics of the corresponding triplet.* Detailed insight into the dynamics is provided in Fig. 2 showing the dependence of photon-pair numbers 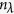 on the Schmidt coefficients 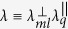 for several pump powers *P* forming an increasing succession. For high powers *P*, the pump modes with the largest Schmidt coefficients *λ* (corresponding to the lowest-order modes with small numbers *l* and *q*) are completely depleted inside the crystal and they even take some energy back from their signal and idler modes contrary to the rest of pump modes (with lower Schmidt coefficients *λ*), which are only losing their energy during the propagation in the crystal. This results in the slower-than-exponential growth of the signal- and idler-field intensities. The observed nearly-linear character of this growth is a consequence of the large number of modes with different evolution that constitute the TWB (see Fig. 2 for the density 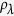 of modes: the smaller the *λ* the larger the density 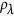). The comparison of curves in Fig. 1 reveals that the GPA slightly underestimates the photon-pair number *N*, which is correctly provided by the SCM for any power *P*. The experimental mean signal-photon numbers 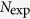 detected in a small portion of the emission PDC cone^18^ are linearly proportional (within the experimental error) to the theoretical photon-pair numbers *N* of both models.

Pump depletion and back-flow of energy occurring in individual modes’ triplets qualitatively influence the coherence of TWB. Both the spatial and spectral intensity auto- and cross-correlation functions widen at the increasing power *P* (see Fig. 3). This is a consequence of the fact that the signal and idler modes with the largest Schmidt coefficients *λ* take the energy from the pump modes much faster than the remaining modes, thus becoming more and more dominant as the power *P* increases (compare the curves for 25 and 35 mW in Fig. 2). Since the phase variation along the spatial and spectral profiles of these highly-populated modes with small numbers *l* and *q* is small compared to the remaining modes [for the profiles, see the Supplementary material], coherence of the TWB naturally increases. This is accompanied by a decrease in the number *K* of modes effectively constituting the TWB. The number *K* of such modes can be quantified, e.g., by the Fedorov ratio^35^ (see Fig. 4).

However, at a certain pump power 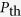, the TWB coherence begins to decrease. At this threshold power 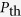, the TWB modes with the largest Schmidt coefficients *λ* took all the power from their pump modes somewhere inside the crystal and so they had to return at least part of it back. This allows the modes with smaller Schmidt coefficients *λ* to become the most populated part of the TWB (see the curve for 100 mW in Fig. 2). This results in the increase of the number *K* of modes accompanied by a partial loss of the spatial and spectral coherence. We note that whereas 40000 spatial and 51 spectral modes constitute the analyzed weak TWB, 1200 spatial and 13 spectral modes are found in the TWB for the threshold power 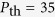 mW.

The curves plotted in Figs 1, 2, 3, 4 have been obtained for a *β* barium borate (BBO) crystal cut for a nearly collinear spectrally degenerate type-I interaction (cut angle 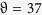 deg), having effective length 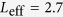 mm and pumped by a beam 530 *μ*m-wide with the central wavelength 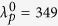 nm and spectrum 1.2-nm wide (see Methods for details about the experiment). The Schmidt modes considered in both models have been determined in^36^ (for more detailed analysis of the modes, see^37^) and applied for un-depleted^17^ as well as depleted^34^ pump beams. These curves fit the experimental points obtained for an 8-mm long BBO crystal measured under the described conditions. The comparison reveals that the model predicts well the behavior of spectral and spatial auto- and cross-correlation functions. Whereas the experimental auto-correlation functions are narrower than the corresponding cross-correlation functions, the model provides very similar profiles for both of them. This originates in the spectral and spatial factorization of modes assumed in the model. The effective crystal length of 2.7 mm used in the model corresponds to the nonlinear walk-off length of the used BBO crystal. For longer crystals, the pump- and TWB modes lose their spatial synchronization due to the walk-off which results in a complex nonlinear interaction in the transverse plane. Distortion of the pump-beam transverse profile occurs^18^. The comparison of graphs in Figs 3 and 4 reveals that the model is still able to predict the TWB behavior in its spectral part, but it underestimates the spatial coherence and, hand by hand, overestimates the number of spatial modes. Importantly, this comparison clearly shows that *the generalized parametric approximation* gives practically the same intensity auto- and cross-correlation functions as well as numbers of modes of TWBs compared to the semiclassical model. This justifies the use of the GPA for the determination of coherence properties of the TWBs for the considered pump powers *P*.

Detailed analysis of the model based on the graph in Fig. 2 points out the occurrence of more threshold powers 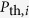 at which the TWB coherence exhibits local maxima. Indeed, these local maxima are observed for powers *P* at which the modes with the largest Schmidt coefficients 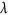 attain their maximal populations (see the curve for 390 mW in Fig. 2). Note that, in the TWB there occur 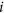 differently-populated groups of modes for an 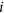-th threshold power 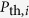 (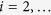). As a result, coherence maxima may be less resolved for larger values 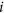. Also, in accord with the previous findings, the number 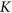 of modes is substantially reduced for these powers 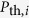.

The developed theory of intense PDC may easily be applied to other nonlinear structures including poled nonlinear materials and waveguiding structures. Similar approaches can be developed to describe other nonlinear interactions including triplets of mutually interacting fields. Raman and Brillouin scattering can be mentioned as typical examples. For all these processes, the relation between the internal structure and coherence of the interacting fields would provide a completely new insight into the evolution of the nonlinear interaction.

In conclusion, we have developed a theory for intense twin beams applicable in the regime of pump depletion. Following the dynamics of individual modes’ triplets, the theory naturally explains the experimentally observed increase (decrease) of spatial and spectral coherence accompanied by a decrease (increase) of the number of modes observed in different regimes of pump powers. The comparison of the results with the experimental data and with the results of semiclassical model confirms the validity of the suggested *generalized parametric approximation* for treating intense twin beams. The model also predicts the occurrence of additional coherence maxima for high pump powers. This represents a challenge for further experimental investigations of intense twin beams.

## Methods

### Experimental setup

The experiment^18^ was performed in a setup shown in Fig. 5(a) using a type-I 8-mm long BBO crystal (cut angle = 37 deg). The crystal was pumped by the third-harmonic pulses (349 nm, 4.5-ps pulse duration) of a mode-locked regeneratively amplified Nd:YLF laser (High-Q-Laser) running at 500 Hz. Radial profile of the pump beam, collimated by means of a telescope in front of the crystal, was 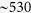 *μ*m wide (FWHM) at the lowest pump power. A half-wave plate followed by a polarizing-cube beam splitter was used to change the pump power. Phase-matching in the crystal for frequency degenerate down-converted beams was reached in a slightly non-collinear configuration. The down-converted light was collected by a lens with 60-mm focal length and then focused in the plane of the vertical slit of an imaging spectrometer (Lot Oriel, 600 lines/mm grating). The angularly dispersed far-field radiation was recorded, shot by shot, by a synchronized EMCCD camera (iXon Ultra 897, Andor) operated at full-frame resolution (512 × 512 pixels, 16-*μ*m pixel size). We note that for the detection of intense TWBs EMCCD cameras are more convenient compared to iCCD cameras, which have been often exploited in this area^38^,^39^,^40^,^41^. The overall resolution of the system composed of the imaging spectrometer and the camera was 0.2 nm in frequency and 0.015 deg in the radial angle. The presence of intensity correlations between the signal and idler beams was verified by observing symmetrically-positioned speckles around the degenerate wavelength and the collinear direction, as shown in Fig. 5(b). Whereas the spectral correlations are observed in the horizontal directions, the spatial radial correlations are visible in the vertical direction in Fig. 5(b).

### Image processing

The intensity auto- and cross-correlation functions characterizing the TWB coherence are obtained as correlation coefficients between a single pixel (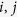) and all the pixels (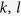) contained in a single-shot image:


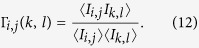


**Figure 1:**
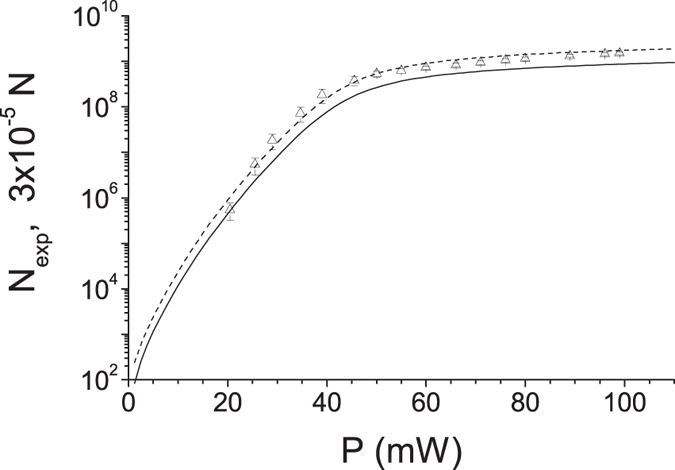
Numbers 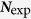 of experimental signal photons (Δ with error bars) detected in a small area of the emission PDC cone measured for different pump powers 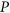
^18^. Numbers 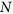 of emitted photon pairs as obtained in GPA (solid curve) and SCM (dashed curve) are shown for comparison; 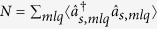.

**Figure 2:**
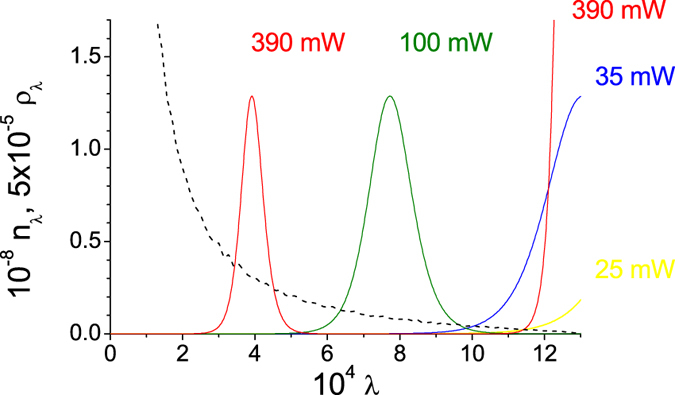
Numbers 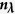 of photon pairs in modes with the Schmidt coefficients 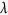 for pump powers *P*  = 25 mW (yellow solid curve), 35 mW (blue), 100 mW (green) and 390 mW (red). Density 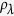 of spatio-spectral modes revealed by the Schmidt decomposition is also shown (dashed curve); 
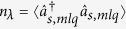
 for 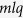 such that 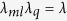.

**Figure 3:**
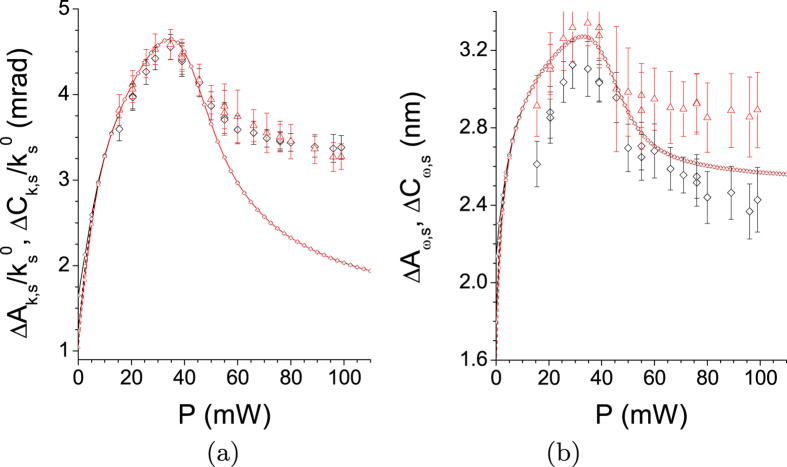
(**a**) Spatial radial and (**b**) spectral widths 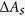 and 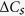 of intensity auto- (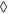, black) and cross-correlation (Δ, red) functions, respectively, versus pump power *P*; experiment (isolated symbols with error bars^18^), GPA (solid curves with symbols), SCM (dashed curves with symbols); 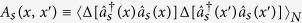, 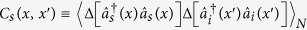, 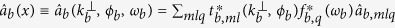 and 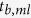 (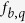) are the spatial (spectral) Schmidt modes of field *b*, 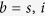. For more details, see^17^. All 4 curves in (**a**) and (**b**) nearly coincide.

**Figure 4:**
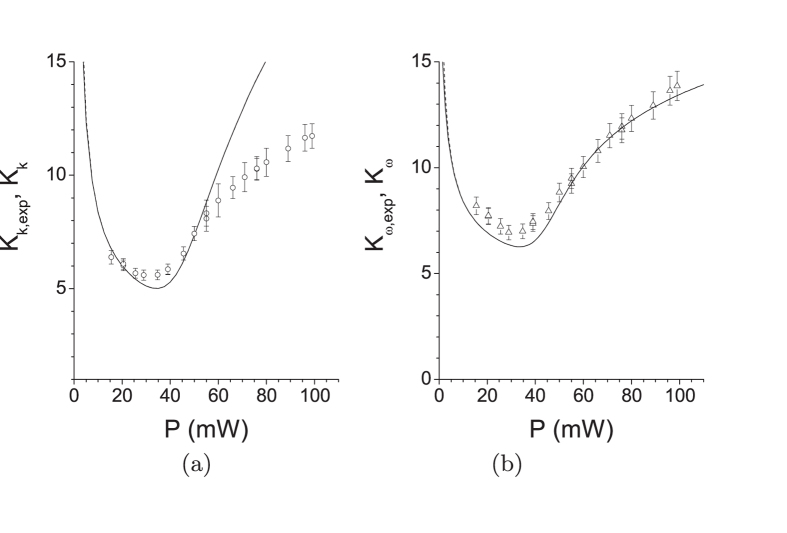
(**a**,**b**) Numbers 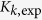 [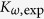] of spatial radial [spectral] modes (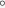 [Δ] with error bars) experimentally detected in a small area inside the emission PDC cone as functions of pump power *P*^18^. For comparison, suitably rescaled spectral (spatial radial) Fedorov ratios 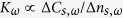 (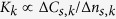) obtained in GPA (solid curve) and SCM (dashed curve) are also drawn. The theoretical curves nearly coincide in both cases.

**Figure 5:**
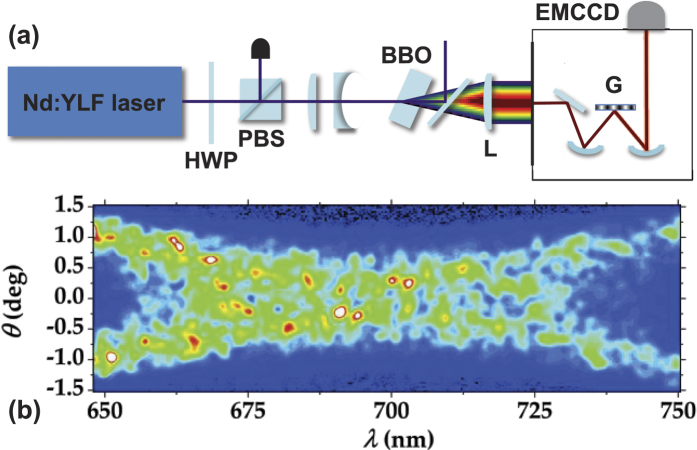
(**a**) Experimental setup used for the determination of spatio-spectral intensity correlations in a TWB. HWP: half-wave plate; PBS: polarizing cube beam splitter; BBO: nonlinear crystal; L: lens, with 60-mm focal length; M_*j*_: spherical mirrors; G: grating; EMCCD: electron-multiplying CCD camera. (**b**) A typical single-shot image of the output of the imaging spectrometer. The signal (positive values) and idler (negative values) spatial radial emission angle *θ* versus the wavelength *λ* is plotted. The speckle-like pattern with correlated grains is visible.

In (12), 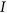 stands for the intensity value of each pixel expressed in digital numbers and after subtraction of the mean value of the noise measured with the camera in perfect dark. Symbol 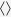 indicates the averaging over a sequence of 1000 subsequent images taken in the experiment. This calculation results in a matrix of the same size as that of the single-shot images. In this matrix, two peaks occur. One peak characterizes the auto-correlation area, the other peak describes the cross-correlation area^41^. Error bars shown in Figs 1, 2, 3, 4 in the main text were obtained by performing the calculation for different pixels. The mean photon numbers have been obtained from a small area (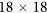 pixels) close to frequency degeneracy and in quasi-collinear interaction geometry. Calibration of the camera sensitivity, its quantum efficiency and all the optical losses were taken into account.

The number of TWB modes in the radial direction was determined by the Fedorov ratio^35^ defined as the ratio between the radial signal-field intensity width and the width of radial intensity cross-correlation function. On the other hand, as the fields’ spectral widths were not measured, the 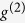 intensity auto-correlation function^18^,^33^,^42^ was used to quantify the number 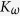 of spectral modes (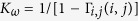).

## Additional Information

**How to cite this article**: Peřina, J. *et al.* Internal dynamics of intense twin beams and their coherence. *Sci. Rep.* **6**, 22320; doi: 10.1038/srep22320 (2016).

## Supplementary Material

Supplementary Information
