# Supplementary material for: Internal dynamics of intense twin beams and their coherence
Source: Sci Rep. 2016 Feb 29;6:22320. doi: 10.1038/srep22320 (PMC4770431; doi:10.1038/srep22320)
Supplement: Supplementary Information [file srep22320-s1.pdf]

Supplemental material: Internal dynamics of intense twin beams and their coherence

Jan Peřina Jr.[1,*], **Ondřej Haderka**[2], **Alessia Allevi**[3,4], **and Maria Bondani**[5,4]

[1]RCPTM, Joint Laboratory of Optics of Palacký University and Inst. Phys. CAS, 17. listopadu 12, 77146 Olomouc, Czech Republic
[2]Institute of Physics CAS, Joint Laboratory of Optics, 17. listopadu 50a, 77146 Olomouc, Czech Republic
[3]Dipartimento di Scienza e Alta Tecnologia, Università degli Studi dell'Insubria, Via Valleggio 11, 22100 Como, Italy
[4]CNISM UdR Como, via Valleggio 11, 22100 Como, Italy
[5]Istituto di Fotonica e Nanotecnologie, Consiglio Nazionale delle Ricerche, Via Valleggio 11, I-22100 Como, Italy
[*]jan.perina.jr@upol.cz

ABSTRACT

Derivation of the spatio-spectral Schmidt modes in spontaneous parametric down-conversion is presented assuming rotational symmetry in the transverse plane.

The positive-frequency electric-field amplitudes $E_a^{(+)}$ of the interacting fields occurring in the momentum operator $\hat{G}_{\rm int}$ of parametric down-conversion written in Eq. (1) of the main text can be decomposed into harmonic plane waves:

$$E_a^{(+)}(\mathbf{r},t) = \frac{1}{\sqrt{2\pi}^3} \int d\mathbf{k}_a \, E_a^{(+)}(\mathbf{k}_a) \exp(i\mathbf{k}_a\mathbf{r} - i\omega_a t), \qquad a = p,s,i. \tag{1}$$

In Eq. (1), $\mathbf{k}_a$ denotes a wave vector of mode a with frequency ω_a. In the paraxial approximation, the plane wave with wave vector $\mathbf{k}_a$ is usually described by its frequency ω_a and transverse wave vector $\mathbf{k}_a^{\perp}$.

The signal and idler spectral negative-frequency electric-field amplitudes $\hat{E}_a^{(-)}(\mathbf{k}_a^{\perp},\omega_a)$ $[\hat{E}_a^{(-)} = (\hat{E}_a^{(+)})^{\dagger}]$ are expressed at the quantum level via the appropriate creation operators $\hat{a}^{\dagger}(\mathbf{k}_a^{\perp},\omega_a)$:

$$\hat{E}_a^{(-)}(\mathbf{k}_a^{\perp},\omega_a) = -i\sqrt{\frac{\hbar\omega_a^2}{2\varepsilon_0 c^2 k_a}}\, \hat{a}_a^{\dagger}(\mathbf{k}_a^{\perp},\omega_a). \tag{2}$$

Symbol $\hbar$ stands for the reduced Planck constant and c is the speed of light in vacuum; $k_a = |\mathbf{k}_a|$.

First, we evaluate the spatial Schmidt modes using the mutual signal- and idler-field amplitude correlation function T_L:

$$T_L(\mathbf{k}_s^{\perp},\mathbf{k}_i^{\perp}) = E_p^{\perp}(\mathbf{k}_s^{\perp}+\mathbf{k}_i^{\perp}) \exp\left(-i\left[\frac{|\mathbf{k}_s^{\perp}+\mathbf{k}_i^{\perp}|^2}{2k_p} - \frac{|\mathbf{k}_s^{\perp}|^2}{2k_s} - \frac{|\mathbf{k}_i^{\perp}|^2}{2k_i}\right]\frac{L}{2}\right) \mathrm{sinc}\left(\left[\frac{|\mathbf{k}_s^{\perp}+\mathbf{k}_i^{\perp}|^2}{2k_p} - \frac{|\mathbf{k}_s^{\perp}|^2}{2k_s} - \frac{|\mathbf{k}_i^{\perp}|^2}{2k_i}\right]\frac{L}{2}\right). \tag{3}$$

The correlation function T_L arises from the phase-matching conditions and depends on the pump-field transverse spatial spectrum $E_p^{\perp}(\mathbf{k}_p^{\perp})$. In Eq. (3), $\mathrm{sinc}(x) \equiv \sin(x)/x$, δ stands for the Dirac δ function, $|\mathbf{k}_a^{\perp}|^2 = k_{a,x}^2 + k_{a,y}^2$ and L denotes the crystal length.

To decompose the correlation function T_L into the spatial Schmidt modes,[1,2] we simplify the geometry of nonlinear interaction and assume radial symmetry in the transverse plane. Due to the radial symmetry, the Schmidt modes in the azimuthal direction are the harmonic functions $[\exp(im\varphi_a)/\sqrt{2\pi}]$ and the Schmidt modes in the radial direction $[u_{a,ml}]$ can be numerically evaluated by performing the Schmidt decomposition of appropriate functions depending only on the radial wave-vector coordinates of signal and idler fields (for details, see[3]). Using the spatial Schmidt modes, the correlation function T_L is expressed as

$$T_L(\mathbf{k}_s^{\perp},\mathbf{k}_i^{\perp}) = \frac{t^{\perp}}{2\pi\sqrt{k_s^{\perp}k_i^{\perp}}} \sum_{m=-\infty}^{\infty}\sum_{l=0}^{\infty} \lambda_{ml}^{\perp} t_{s,ml}(k_s^{\perp},\varphi_s) t_{i,ml}(k_i^{\perp},\varphi_i), \tag{4}$$

where

$$t_{s,ml}(k_s^{\perp},\varphi_s)=u_{s,ml}(k_s^{\perp})\exp(im\varphi_s),\qquad t_{i,ml}(k_i^{\perp},\varphi_i)=u_{i,ml}(k_i^{\perp})\exp(-im\varphi_i).$$

In Eq. (4), $\lambda_{ml}^{\perp}$ denote the Schmidt coefficients and $t^{\perp}$ is a normalization constant. We note that, in the considered type-I interaction, the pump field propagates as an extraordinary wave which results in certain anisotropy.[4,5] For simplicity, here we omit this anisotropy.

The dual spatial Schmidt modes $t_{s,ml}$ and $t_{i,ml}$ allow us to simplify the formula for momentum operator $\hat{G}_{\rm int}$ by introducing the following unitary transformations of the field operators,

$$\hat{a}_{a,ml}(\omega_a,z)=\int_0^{\infty}dk_a^{\perp}\int_0^{2\pi}d\varphi_a\,t_{a,ml}^{*}(k_a^{\perp},\varphi_a)\hat{a}_a(k_a^{\perp},\varphi_a,\omega_a,z),\qquad a=s,i. \tag{5}$$

Using these newly defined fields' operators, the first-order perturbation solution of the Schrödinger equation can be written as:

$$|\psi\rangle_{\rm out}=t^{\perp}\sum_{m,l}\lambda_{ml}^{\perp}\int_0^{\infty}d\omega_s\int_0^{\infty}d\omega_i\,F_L(\omega_s,\omega_i)\hat{a}_{s,ml}^{\dagger}(\omega_s,0)\hat{a}_{i,ml}^{\dagger}(\omega_i,0)|{\rm vac}\rangle \tag{6}$$

where $|{\rm vac}\rangle$ stands for the input vacuum state. The two-photon spectral amplitude F_L occurring in Eq. (6) takes the form

$$\begin{aligned}F_L(\omega_s,\omega_i)\;=\;&\frac{2id_{\rm eff}L}{\sqrt{2\pi}^3c^2}\frac{\omega_s\omega_i}{\sqrt{k_sk_i}}E_p^{\parallel}(\omega_s+\omega_i)\exp\left(-i[k_p(\omega_s+\omega_i)-k_s(\omega_s)-k_i(\omega_i)]L/2\right)\\&\times\,{\rm sinc}\left([k_p(\omega_s+\omega_i)-k_s(\omega_s)-k_i(\omega_i)]L/2\right).\end{aligned} \tag{7}$$

Symbol $E_p^{\parallel}$ denotes the pump-field spectrum and $d_{\rm eff}$ is an effective nonlinear coupling constant ($d_{\rm eff}=\chi^{(2)}/2$). The spectral Schmidt decomposition of two-photon amplitude F_L is written as follows

$$F_L(\omega_s,\omega_i)=f^{\parallel}\sum_{q=0}^{\infty}\lambda_q^{\parallel}f_{s,q}(\omega_s)f_{i,q}(\omega_i) \tag{8}$$

using the spectral Schmidt coefficients $\lambda_q^{\parallel}$ and the Schmidt mode functions $f_{s,q}$ and $f_{i,q}$; $f^{\parallel}$ denotes an appropriate normalization constant. The introduction of new field operators $\hat{a}_{a,mlq}$,

$$\hat{a}_{a,mlq}=\int_0^{\infty}d\omega_a\,f_{a,q}^{*}(\omega_a)\hat{a}_{a,ml}(\omega_a,0),\qquad a=s,i, \tag{9}$$

results in the output state $|\psi\rangle_{\rm out}$ written in the following simple form:

$$|\psi\rangle_{\rm out}=t^{\perp}f^{\parallel}\sum_{m,l,q}\lambda_{ml}^{\perp}\lambda_q^{\parallel}\hat{a}_{s,mlq}^{\dagger}\hat{a}_{i,mlq}^{\dagger}|{\rm vac}\rangle. \tag{10}$$

According to formula (10) the output state $|\psi\rangle_{\rm out}$ is given as a quantum superposition of independent paired spatial and spectral modes. We note that there may occur degeneracy in which the signal and idler modes coincide.[6] However, in the considered non-collinear interaction geometry, the contribution of such modes is negligible.

The number of transverse modes is usually large and so their Schmidt coefficients $\lambda_{ml}^{\perp}$ form quasi-continuum. As a consequence, we may replace the sum over transverse modes by the following integral using a suitable probability function ρ_λ:

$$\sum_{ml}\longrightarrow\int_0^{\lambda_{\rm max}^{\perp}}d\lambda^{\perp}\rho_\lambda(\lambda^{\perp}); \tag{11}$$

$\lambda_{\rm max}^{\perp}$ equals to the largest Schmidt transverse coefficient $\lambda_{ml}^{\perp}$.

To illustrate the behavior of spectral and spatial Schmidt modes, we consider a BBO crystal 2.7-mm long under the conditions described in the main text. We plot in Fig. 1 their intensity profiles for the first three spectral modes $f_{s,q}$ and the first three radial modes $u_{s,ml}$ for the azimuthal number $m=0$. In general, a q-th (l-th) spectral (spatial radial) mode has $q-1$ minima in its profile. Also, the larger the number q (l) the more extended the mode profile is. The probability function ρ_λ of the Schmidt transverse coefficients and the Schmidt spectral coefficients $\lambda_q^{\parallel}$ for the considered crystal are plotted in Fig. 2. More details can be found in.[7]

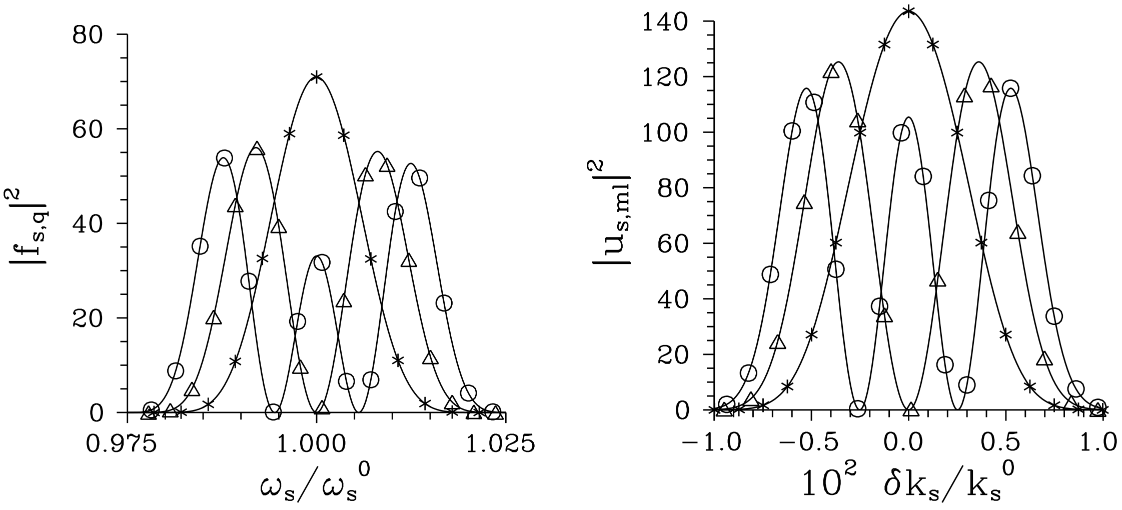

(a) (b)

Figure 1. (a) Spectral intensity profiles $|f_{s,q}|^2$ of the Schmidt modes for $q = 0$ (solid curve with $*$), $q = 1$ (solid curve with $\triangle$) and $q = 2$ (solid curve with $\circ$) and (b) spatial radial intensity profiles $|u_{s,0l}|^2$ of modes for $l = 0$ (solid curve with $\triangle$), $l = 1$ (solid curve with $*$) and $l = 2$ (solid curve with $\circ$) in the signal field. Normalization is such that $\int d\omega_s |f_{s,q}(\omega_s)|^2/\omega_s^0 = 1$ and $\int dk_s |u_{s,0l}(k_s)|^2/k_s^0 = 1$.

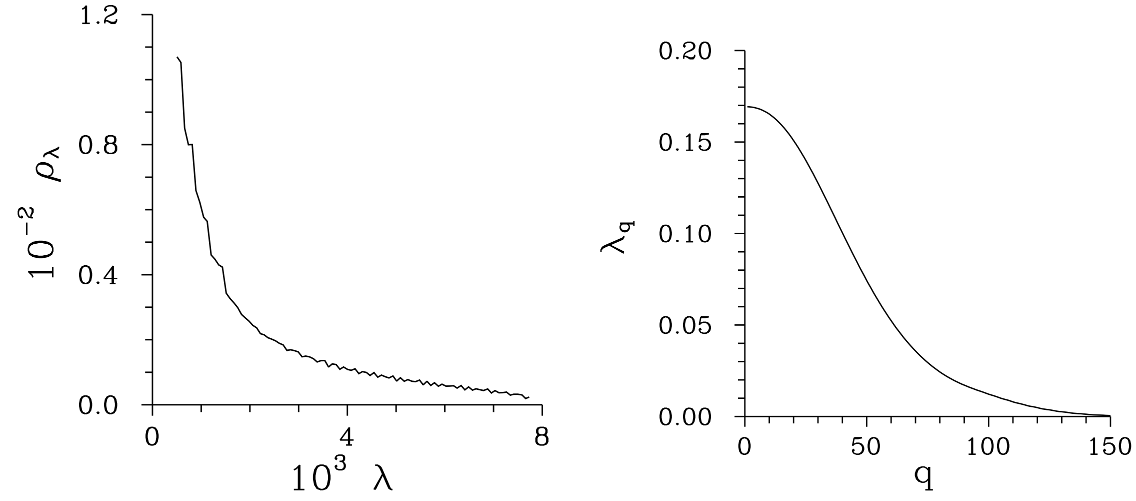

(a) (b)

Figure 2. (a) Probability function ρ_λ of the Schmidt transverse coefficients $\lambda^{\perp}_{ml}$ and (b) the Schmidt spectral coefficients $\lambda^{\parallel}_{q}$.

References

1. Law, C. K., Walmsley, I. A. & Eberly, J. H. Continuous frequency entanglement: Effective finite Hilbert space and entropy control. *Phys. Rev. Lett.* **84**, 5304—5307 (2000).

2. Law, C. K. & Eberly, J. H. Analysis and interpretation of high transverse entanglement in optical parametric down-conversion. *Phys. Rev. Lett.* **92**, 127903 (2004).

3. Peřina Jr., J. Coherence and dimensionality of intense spatiospectral twin beams. *Phys. Rev. A* **92**, 013833 (2015).

4. Fedorov, M. V. *et al.* Spontaneous parametric down-conversion: Anisotropical and anomalously strong narrowing of biphoton momentum correlation distributions. *Phys. Rev. A* **77**, 032336 (2008).

5. Fedorov, M. V. *et al.* Anisotropical and high entanglement of biphoton states generated in spontaneous parametric down-conversion. *Phys. Rev. Lett.* **99**, 063901 (2007).

6. Fedorov, M. V. & Miklin, M. I. Schmidt modes and entanglement. *Contemporary Phys.* **55**, 94—109 (2014).

7. Peřina Jr., J. Coherence and mode decomposition of weak twin beams. *Phys. Scr.* **90**, 074058 (2015).
